# The impact of immigration detention on children’s mental health: systematic review

**DOI:** 10.1192/bjp.2025.29

**Published:** 2025-12

**Authors:** Isabella Priestley, Sarah Cherian, Georgia Paxton, Zachary Steel, Peter Young, Hasantha Gunasekera, Caroline Hunt

**Affiliations:** School of Psychology, The University of Sydney, Sydney, Australia; Refugee Health Service, Department of General Paediatrics, Perth Children’s Hospital, Nedlands, Australia; Division of Paediatrics, University of Western Australia, Perth, Australia; The Kids Research Institute, Nedlands, Australia; Immigrant Health, Department of General Medicine, Royal Children’s Hospital, Melbourne, Australia; Infection and Immunity, Murdoch Children’s Research Institute, Parkville, Australia; Psychiatry and Mental Health, University of New South Wales, Sydney, Australia; Independent researcher, Sydney, Australia; Children’s Hospital Westmead Clinical School, The University of Sydney, Sydney, Australia

**Keywords:** Trauma, mental health, childhood, immigration detention, refugee health

## Abstract

**Background:**

There are 117.3 million people forcibly displaced because of war, conflict and natural disasters: 40% are children. With growing numbers, many high-income countries have adopted or are considering increasingly restrictive policies of immigration detention. Research on the impact of detention on mental health has focused on adults, although recent studies report on children.

**Aims:**

To synthesise data on the impact of immigration detention on children’s mental health.

**Method:**

Systematic searches were conducted in PsycINFO, MEDLINE and Embase databases and grey literature and studies assessed using PRISMA guidelines (PROSPERO registration CRD42023369680). Included studies were quantitative, assessed children younger than 18 years who had been in immigration detention and reported mental health symptoms or diagnoses. Methodological quality was assessed using the Appraisal Tool for Cross-Sectional Studies. Meta-analyses estimated prevalence for major depression and post-traumatic stress disorder (PTSD).

**Results:**

Twenty-one studies reported data on 9620 children. Most studies were cross-sectional, had small sample sizes and used convenience sampling. A profoundly detrimental impact on children’s mental health across a variety of countries and detention settings was demonstrated. Meta-analysis found pooled prevalence of 42.2% for depression [95% CI 22.9, 64.3] and 32.0% for PTSD [95% CI 19.4, 48.0]. Severity of mental health impact increased with exposure to indefinite or protracted held detention.

**Conclusions:**

Immigration detention harms children. No period of detention can be deemed safe, as all immigration detention is associated with adverse impacts on mental health. Our review highlights the urgency of alternative immigration policies that end the practice of detaining children and families.

The United Nations High Commission for Refugees (UNHCR)^1^ estimates 117.3 million people have been forcibly displaced because of war, conflict, persecution and natural disasters. Approximately 40% of this cohort are younger than 18 years of age. Global displacement has more than doubled since 2012^1^ and continues to grow. Forced migration typically results in children and their families being exposed to multiple traumatic events pre-displacement, during the period of flight and post arrival that cumulatively increase mental health risk.^2^

Increasingly, people seeking asylum, including children, are subject to administrative detention as part of immigration processing systems established by reception countries. These detention settings may be high-security facilities and can be located within or outside the borders of reception countries. The Global Detention Project^3^ has documented the expansion of immigration detention facilities, with approximately 2600 immigration detention facilities operating across 198 countries, and 285 new facilities within the last 12 months. Australia’s policy of mandatory indefinite detention, enforced since 1992, occupies a unique position within the global detention framework.^4^

The UNHCR defines immigration detention as ‘confinement within a narrowly bounded or restricted location…where freedom of movement is substantially curtailed and where the only opportunity to leave this limited area is to leave the territory’.^5^ Immigration detention models vary between countries and include holding people seeking refuge in combinations of (a) an open or closed facility, (b) for short or prolonged periods or (c) for definite or indefinite periods. There are no standards to define ‘short’ or ‘prolonged’ time periods, with individual governments determining length of detention. The conditions of immigration detention facilities across high-income reception countries such as the UK,^6^ Australia^7^ and the USA^8^ have been described as overcrowded, unhygienic and unsafe. For children, the detention environment is developmentally inappropriate, with limited opportunities for them to engage freely in play and recreation.^9–11^ The Australian Human Rights Commission^7^ concluded that the Australian detention environment was extremely dangerous for children, documenting numerous reports of physical and sexual assault involving children. Sexual assaults, mostly involving children, were reported on average every 13 days in Australian immigration detention centres. In Australian immigration detention, children were also living in close confinement with adults experiencing high levels of mental distress (e.g. witnessing adult self-harm and suicide attempts) and were at risk of experiencing neglect or maltreatment by parents, other detainees struggling with poor mental health and from staff.^7^ In some centres (e.g. Christmas Island) children had limited or no access to school. In Australian detention on the Republic of Nauru, children of varying ages were taught together in a single school for 2 years before being sent to local schools that were not equipped to provide education to this cohort.^7,12^ Most children and their families were detained for a prolonged and indefinite period, often following trauma before detention, leading to widespread mental health difficulties and feelings of hopelessness and despair.^7,13^

Adverse childhood experiences (ACEs),^14^ especially exposure to violence,^15^ are well-established risk factors for the development of mental health disorders that can persist into adulthood. Children in detention are also vulnerable given their reliance on adult caregivers, who may themselves be experiencing the effects of detention, influencing their ability to provide the nurturance needed for their child’s healthy development.^11,16^ Children’s mental health may not only be affected by an impoverished detention environment, but also the disruption to family cohesion and functioning, including family separations, and exposure to unrelated adults experiencing distress and mental illness. Detention is a recognised adverse experience for children from refugee-like backgrounds.^17^

It is difficult to estimate the current number of children being held in immigration detention globally, as many governments do not publish such information, and there is typically limited independent oversight of detention centres. The quality of data reported by governments may also be questionable. For example, before 2014, mental health assessments of children held in Australian immigration detention were not conducted using a child-specific measurement tool, and data regarding the mental health of children were not disaggregated from adult data.^7^ Research regarding the impact of immigration detention on mental health has been historically difficult to conduct because of a lack of access to these cohorts in reception countries. Where access has been possible, researchers have faced multiple ethical dilemmas, such as whether true informed consent is possible given people seeking asylum have high levels of mental distress and vulnerabilities regarding their visa status,^18,19^ with no research on children’s consent/assent in this setting.

Previous systematic reviews^11,20–22^ have found mental health difficulties are pronounced in people held in immigration detention, with common findings across a variety of ethnic groups and reception countries, including Australia, the UK and the USA, in predominantly adult cohorts. Several studies^23,24^ have found that the experience of immigration detention has an independent effect on the mental health of people seeking asylum, distinguishable from previous trauma and stressors. The experience of immigration detention may exacerbate pre-existing vulnerabilities and/or contribute to new and ongoing mental health difficulties.^11^

In comparison to research in adults, fewer studies have examined the mental health of children held in immigration detention. Verhülsdonk et al’s systematic review and meta-analysis^21^ included only 26 children in two studies, reporting high prevalence of depression (68%), anxiety (54%) and post-traumatic stress disorder (PTSD) (42%). Von Werthern et al^11^ included 629 children in nine studies, although prevalence of mental health difficulties is derived from 51 children in three studies, and Robjant et al^20^ included 40 children in two studies and a participant observer account. Previous systematic reviews^11,20,21^ have not included grey literature and have employed strict inclusion criteria, which may have excluded relevant studies (e.g. from European reception centres with less stringent detention policies but that may still detain children for extended periods).

The current review is timely. Several new studies have been published in the last few years examining the mental health of children held in immigration detention. Furthermore, countries including the UK^25^ and Denmark^26^ are considering the implementation of more restrictive detention policies modelled on Australia’s mandatory detention policies, such as the removal of time limits for detention or offshore processing arrangements, and the use of detention is expanding globally. This systematic review aims to provide an updated synthesis of evidence on the impact of immigration detention on children’s mental health to determine mental health burden, inform policymakers and ensure children are adequately considered in asylum resettlement programmes.

## Method

### Search strategy and selection criteria

We followed PRISMA 2020 guidelines for systematic reviews using a context, condition, population framework (CoCoPop).^27^ The condition of interest was mental health symptoms or diagnoses, including impacts on physical and developmental health. The context of interest was immigration detention. The population of interest was children younger than 18 years.

Studies were eligible for inclusion if they were published in English and reported on all the following: (a) population younger than 18 years; (b) held in immigration detention; (c) mental health symptoms/disorders; and (d) included quantitative data. A focus on quantitative studies allowed examination of mental health data across different systems of detention. A formal synthesis of qualitative studies was deemed beyond the scope of the current review. The protocol was preregistered in the International Prospective Register of Systematic Reviews (PROSPERO; registration number: CRD42023369680).

Relevant studies were identified using electronic searches of PsycINFO (1806 to Week 29, July 2023) Ovid MEDLINE (1946 to 17 July 2023) and Embase (1974 to 17 July 2023) databases. Search terms related to the following four areas: mental health; immigration detention; children; and refugees/asylum seekers/migrants (see Supplementary Table 1). All search results were downloaded to EndNote™ (X21 Clarivate, St Helier, Jersey; see https://endnote.com/?srsltid=AfmBOoozClzSKkfd2sqlKbpIxHuYJ3WLs3lXHxUxKMoQ028RqsS560e7) and exported to Covidence™ (2025 Covidence, Melbourne, Australia; see https://www.covidence.org/).

One reviewer (I.P.) reviewed the titles and abstracts of studies identified from the electronic search against the inclusion and exclusion criteria. Duplicate results were excluded, including Zwi et al,^28^ which was a duplicate subset of the Mares study.^18^ Full texts were retrieved if eligibility was unclear. Two reviewers (I.P./C.H.) independently assessed the full texts of all potentially relevant studies. Disagreement on eligibility was resolved by reviewer discussion with a third reviewer. This search strategy was supplemented by a hand-search of the following specialist journals published between 2018 and 2023: *Forced Migrant Review*, *International Migration Review*, *International Migration*, *Journal of Refugee Studies*, *Journal of Traumatic Stress, Refuge and Torture*. A search of grey literature was also used to identify reports addressing children in detention published by national governments, national statutory authorities, United Nations bodies (Office of the United Nations High Commissioner for Human Rights (OHCHR), United Nations High Commissioner for Refugees (UNHCR), UNICEF) and non-governmental organisations (Human Rights Watch, Amnesty International, Physicians for Human Rights, Oxfam, Médecins Sans Frontières (MSF), International Organisation for Migration, Save the Children, International Detention Coalition).

The reference lists of all eligible studies and previous systemic reviews and meta-analyses were also reviewed to identify additional studies. Content expert authors with publications in this area (S.C., G.P., Z.S., P.Y. and H.G.) contributed additional papers.

As analysis was based on review of peer-reviewed papers and publicly available reports, no additional human ethical review process was required. This review received no external funding.

### Data extraction

Data from all eligible studies were independently extracted by reviewers (I.P. and C.H.) into an electronic spreadsheet. The following data were extracted from each study: first author; year of publication; study design; description of sample and sampling method; and sample size. The following data on children and settings were extracted: type of detention (held/non-held; short/prolonged/protracted; indefinite/definite); gender; age; year/s of assessment; time spent in detention; country of origin; reception country; outcomes of interest; children witnessing violence in detention; parental mental health; family separation; disruption to schooling; relocations; method of assessment; prevalence of mental health symptoms and diagnoses; and developmental and physical health concerns. Where reported, clinical cut-off score, range, median score, mean score and standard deviation were extracted for each clinical measure. Where relevant, authors were contacted to clarify type of detention, location of detention centre, ages of children detained and time spent in detention.

### Data synthesis, analysis and quality assessment

Extracted data are presented in supplementary tables. Type of detention was categorised as ‘held detention’, a locked centre where detainees were unable to leave; ‘non-held detention’, a centre or setting where detainees could leave; ‘short detention’, an average length of detention less than 1 month; **‘**prolonged detention’, an average length of detention between 1 and 12 months; ‘protracted detention’, an average length exceeding 12 months; and ‘indefinite detention’ processing arrangements where people seeking asylum were detained with no limit specified. We also specified ‘remote’ as located outside metropolitan or regional areas with limited access to physical health, mental health, legal or social support. Severity of detention was ranked by type (most to least restrictive) and duration, with additional weighting given to indefinite detention arrangements (see Supplementary Table 2). Studies were also grouped by nation and region for reporting.

Meta-analysis of the prevalence of mental health disorders was conducted using Comprehensive Meta-Analysis version 4.0 (Biostat Inc., Englewood, NJ, USA; see https://meta-analysis.com/?srsltid=AfmBOoq51ysyePg2sz_E-zlemlXRBBRlSALnr6u6vcJuKG7DsRk2Wy5n), employing the event proportion of positive diagnoses as the effect size index and using a random-effects model to account for data heterogeneity. We report mean prevalence, confidence interval (95% CI), *Q* (test for homogeneity of effect sizes across studies) and *I* ^2^ (measure of the magnitude of heterogeneity, or the percentage of the observed variance that is real rather than spurious). Forest plots were developed to model population effect size. Publication bias or certainty of evidence could not be reliably assessed because of the small number of studies available.

The methodological quality of eligible studies was assessed independently by two reviewers (I.P. and C.H.) using the Appraisal Tool for Cross-Sectional Studies (AXIS)^29^ and discrepancies were resolved by discussion.

## Results

**Fig. 1:**
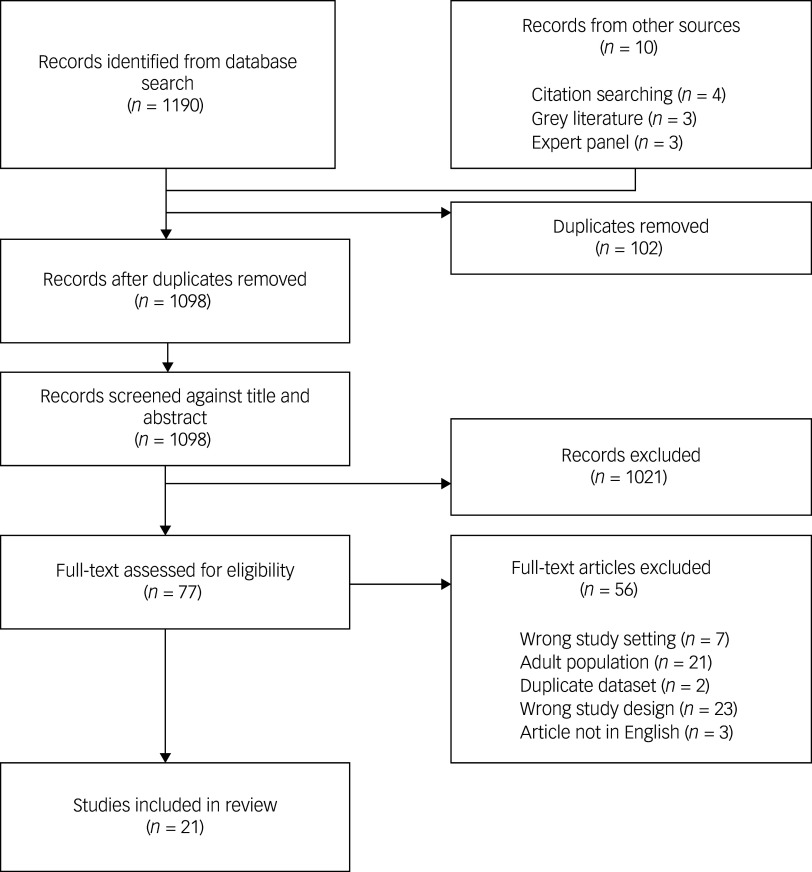
PRISMA flow diagram displaying search results.

The study screening and selection process is outlined in Fig. 1. The electronic search identified 1190 articles; ten additional articles were identified by searching the previous review reference lists (*k* = 4), grey literature search (*k* = 3) and through the expert panel (*k* = 3). After 102 duplicates were removed, 1098 articles were manually screened by review of the title/abstract: 1021 did not meet inclusion criteria. After full-text review of the remaining 77 articles, 21 studies met inclusion criteria.

### Characteristics of included studies

These 21 studies included a total of 9620 children (39.4% female). Most studies were cross-sectional (*k* = 14) and reported the mental health status of detained children examined at a single time point. Nine studies reported on children held in Australian immigration detention, in facilities on the Australian mainland or offshore (Christmas Island, Nauru, Manus Island in Papua New Guinea (PNG)). The remaining 12 studies reported on children held in detention centres in the USA, the UK, Norway, Finland, the Netherlands, Denmark and Libya. Full details of included studies are in Supplementary Table 3.

#### Australian detention facilities (including those established for offshore processing)

Within the Australian context, most children experienced held, prolonged or protracted and indefinite detention on mainland Australia or offshore detention centres (including those used for regional processing in Nauru and PNG). Children were typically detained for a period of several months to years. Two studies^30,31^ included children and families held in the Nauru Regional Processing Centre beyond October 2015, when it transitioned from being a closed/locked facility to an ‘open centre’.^32^ Independent reports indicate that regardless of this policy change, people held in this setting experienced restricted freedom of movement and were unable to leave Nauru, a tiny remote island in the middle of the Pacific Ocean. Two studies^12,31^ included children in non-held, indefinite ‘community detention’, a form of detention used after release from held detention in Australia. Children and their families in community detention were still subject to significant restrictions of movement, schooling, employment, housing and health service access. Most children within Australian detention originated from Iraq, Iran, Afghanistan, Sri Lanka, Somalia and Myanmar (Burma). Some families had babies born in held or community detention.

#### English detention facilities

In two studies in the UK^33,34^ children experienced held, indefinite detention ranging from a short to prolonged periods. The most frequent countries of origin were Afghanistan, Iran, Democratic Republic of Congo, Nigeria, Uganda, Pakistan and Jamaica.

#### USA detention facilities

One study included children in short, held, definite detention at the USA–Mexico border, for an average of 9 days.^35^ The most frequent countries of origin included Honduras, El Salvador and Guatemala. Older studies^36,37^ reported on children in held, prolonged, indefinite detention in Guantanamo Bay Camp for a period of 4–8 months. Data regarding country of origin or mean length of stay were not available.

#### Countries in European Union detention facilities

In most European Union countries (Denmark, the Netherlands and Finland) children and families were generally placed in non-held detention, meaning they had greater freedom of movement.^38–41^ Only one European Union study^42^ (the Netherlands) included a sample of children in held detention. Length of detention in European Union centres ranged from short (0–10 days) to protracted (91 months) periods. The most frequent countries of origin included Syria, Russia, Afghanistan, Iran, Somalia and Iraq, although this varied according to data collection timeframes.

One study^43^ examined unaccompanied minors in held, indefinite detention in centres near Tripoli, Libya. We considered this as a European Union study as the detention centre was financed by the European Union as an offshore facility aimed at preventing Mediterranean crossings. Data regarding time spent in detention were not available. The most frequent countries of origin were Eritrea and Somalia.

### Clinical assessment tools

The most common assessment measure was a clinical interview, which was used in nine studies^30,31,33,34,38,43–46^ and used exclusively in six studies.^30,31,38,44–46^ Validated symptom measures included the Strengths and Difficulties Questionnaire (SDQ) (*k* = 4), the Hopkins Symptom Checklist (*k* = 3), the Reactions of Adolescents to Traumatic Stress (RATS) scale (*k* = 3), the Post-Traumatic Stress Disorder Reactive Index (PTSDRI) (*k* = 3), the Harvard Trauma Questionnaire (HTQ) (*k* = 1) and the Child Trauma Screening Questionnaire (CTSQ) (*k* = 1). Only two studies^33,47^ used validated semi-structured diagnostic interviews (the Schedule for Affective Disorders and Schizophrenia for School-Aged Children-Present and Lifetime Version (K-SADS-PL) and the Structured Clinical Interview for DSM-IV^48^ (SCID-IV)).

### Type, severity, duration and characteristics of detention experience

**Fig. 2:**
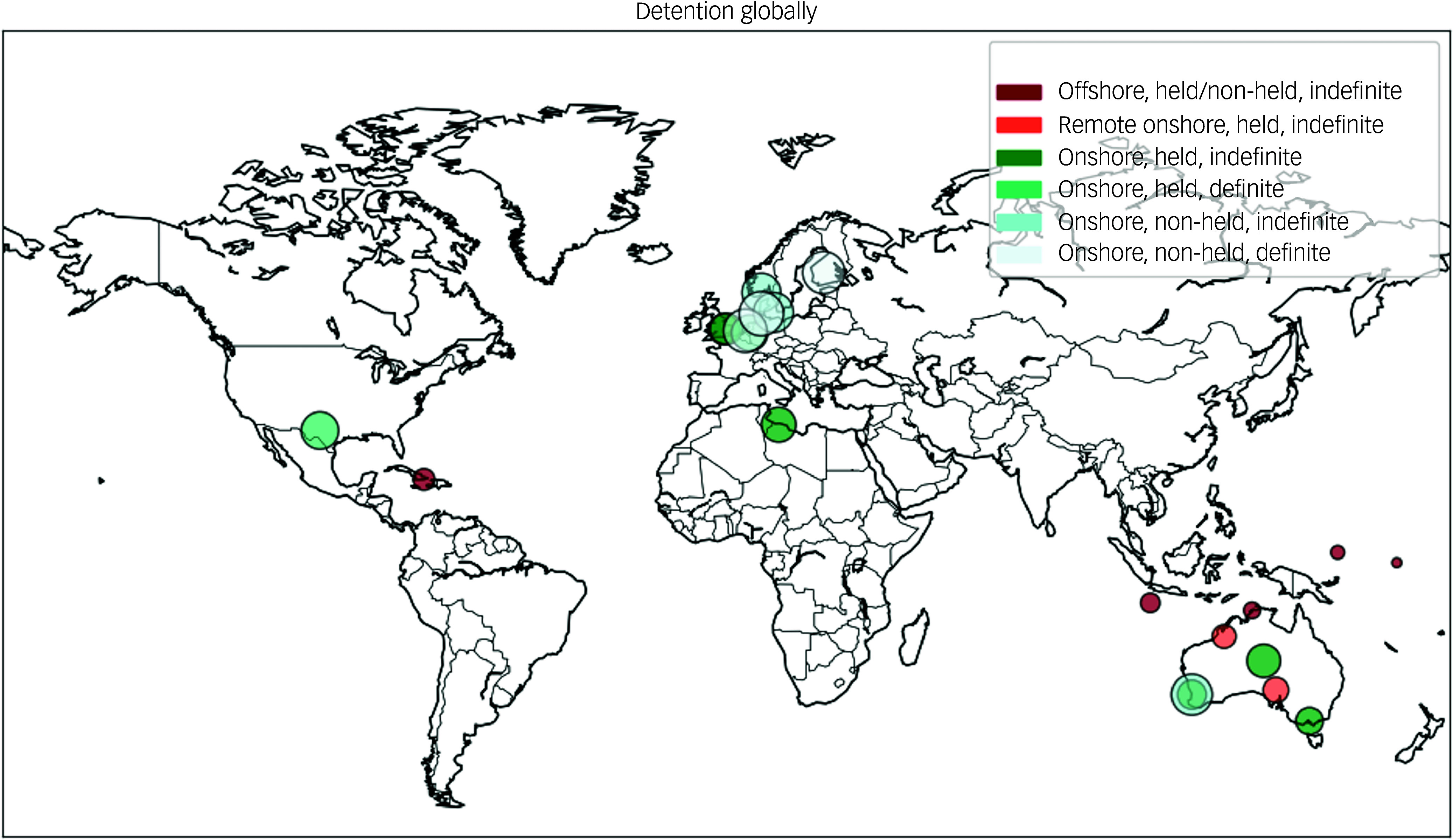
Global map of detention sites in published studies by detention classification.

Studies are ordered according to severity of detention, and then by time spent in detention (Supplementary Table 2). Ten studies reported children witnessing violence; these were mostly children in prolonged, held, indefinite detention. Eight studies identified parental mental health problems, while five studies exclusively sampled unaccompanied minors and were consequently unable to report on parental mental health. Experiences of family separation were reported in 17 studies and had occurred in the context of detention as well as the refugee journey. Thirteen studies reported children experiencing disruption to schooling, including no or partial access to education, delays in enrolment, limited scope of curriculum and children having difficulty concentrating and participating in schoolwork because of mental health problems. Six studies reported children being relocated between detention centres during the period of detention. Poor parental mental health, disruption to schooling and relocations were documented across types of detention, including non-held/definite, non-held/indefinite and held/indefinite. Fig. 2 shows a geographical distribution of samples of children in detention, indicating a predominance of Australian studies examining held, indefinite detention.

### Prevalence of mental health symptoms

Fifteen studies reported on specific mental health symptoms in a total of 8726 children (see Supplementary Table 4). In addition, two studies reported on the prevalence of non-specific psychological distress in their samples. Young and Gordon^49^ found that 34% of 243 children had higher than average mental health scores relative to children attending specialist mental health services when assessed by the clinician-rated Health of the Nation Child and Adolescent Outcome Scores (HoNOSCA). Hanes et al^12^ reported acute/severe psychological concerns in 47% of 109 children as assessed on review of medical records.

#### Anxiety/low mood

Symptoms of anxiety and low mood were reported by three studies,^34,38,44^ all using a clinical interview as the method of assessment. Prevalence of anxiety symptoms ranged from 5% to 100%, and low mood ranged from 2% to 100%. Symptoms of anxiety (5%) and low mood (2%) were uncommon in children held in short, non-held, definite detention.^38^ In comparison, symptoms of anxiety (39–100%) and low mood (47–100%) were frequent in children held in prolonged or protracted, held, indefinite detention.^34,44^

#### Post-traumatic stress

Post-traumatic stress symptoms were reported by six studies. Three studies^35–37^ used the PTSDRI, while others used the HTQ,^39^ RATS scale^33^ and CTSQ.^13^ All children were in held detention at the time of assessment. The prevalence of post-traumatic stress symptoms ranged from 17% to 95%. In studies where children and young people had been held for a short period,^33,35,39^ symptoms ranged from 17% to 80%. The prevalence of post-traumatic stress symptoms was 95% in children who had been held for a prolonged period ranging from 3 to 18 months when assessed in the detention setting.^13,36,37^ Two studies with samples of unaccompanied minors reported post-traumatic stress symptoms of 80%^33^ and 59%.^39^

#### Self-harm

Self-harm behaviours were reported by four Australian studies where children had mostly been held in prolonged, closed, indefinite detention in Australia or Nauru, or in community arrangements on Nauru. Self-harm behaviour was assessed using clinical interviews^31,44,45^ and the K-SADS-PL.^47^ The prevalence of self-harm ranged from 10% to 80%. In one study^44^ data related to self-harm were combined with suicidal ideation and suicide attempts. Tosif et al^31^ found that the prevalence of self-harm was higher in children held in offshore detention on Nauru (27%) compared with detention on mainland Australia (4%). The prevalence of self-harm behaviours in a small sample of children referred to a child and adolescent mental health service (CAMHS) was 80%,^45^ while prevalence was 25% in children from families seeking legal assistance^47^ and 10% in a larger sample of children attending a generalist health service.^31^

#### Suicidal ideation and suicide attempts

Suicidal ideation was reported by three older studies.^41,45,47^ One study reported on combined suicidal ideation/attempts,^36^ and Amarasena et al^44^ reported on combined self-harm and suicidal ideation/attempts as above. All children in these studies were in prolonged, held detention. Prevalence of suicidal ideation ranged from 13% to 100%. Suicidal ideation was reported in 13% of children who had been detained for 4–6 months,^41^ 100% of children detained for 12–18 months^45^ and 55% of children detained for between 2 years and 2 years, 8 months.^45,47^ Suicidal ideation was universal in a sample of children who had been referred to a CAMHS for mental health difficulties.^45^

#### Sleep difficulties

Sleep difficulties were reported in 15–100% and were less frequent in children in short, non-held, definite detention (15%)^38^ compared with children in prolonged or protracted, held or indefinite detention (38–100%).^18,31,34,36,44,45^

#### Strengths and Difficulties Questionnaire data

**Fig. 3:**
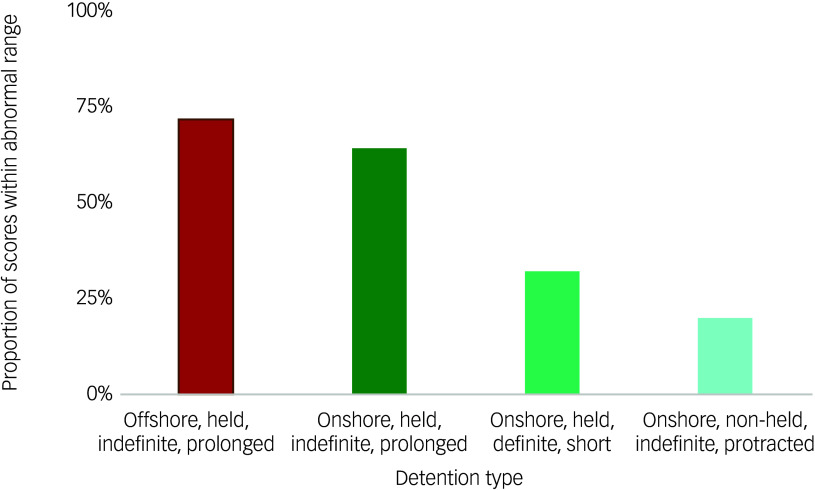
Strengths and Difficulties Questionnaire emotional symptoms subscale scores in children in immigration detention.

Four studies^18,34,35,40^ used the SDQ to assess mental health symptoms (Fig. 3). The prevalence of abnormal scores on the emotional symptoms subscale was 64%^34^ and 72%^18^ for children in prolonged, held, indefinite detention, 32%^35^ for children in short, held, definite detention and 20%^40^ for children in non-held, indefinite, protracted detention.

### Prevalence of physical health issues and developmental concerns

Eleven studies reported on physical and developmental health symptoms, including a total of 7898 children (Supplementary Table 5). Most physical health symptoms were assessed using a clinical interview and examination. Headache prevalence ranged from 8% to 27%; however, two studies combined data related to headache with other somatic complaints: toothache^38^ and nail-biting.^18^ The prevalence of abdominal pain was 16–91%, and that of general somatic concerns was 8–50%. Developmental concerns were identified using clinical interview,^31,34,44,46^ parent reports,^18^ the Parental Evaluation of Developmental Status (PEDS)^13^ or a standardised refugee health screen.^12^ In studies that assessed development, general developmental concerns were reported in 16–100% and problems related to language delay/regression were reported in 4–50% of children.

### Prevalence of mental health disorders

Six studies reported on the prevalence of anxiety disorders, depressive disorders, PTSD and pervasive refusal syndrome (PRS) in a total of 166 children (see Table 1). Three studies included children in Australian offshore detention, three included children in Australian mainland detention centres and one reported data from a UK detention centre. Children in Australian detention centres experienced prolonged or protracted, held and indefinite detention. In comparison, children in the single UK study had experienced short, held, indefinite detention. In four studies,^30,31,44,45^ mental health diagnoses were made using clinical interviews. Two studies used validated semi-structured diagnostic interviews – the K-SADS-PL^47^ and the SCID-IV.^33^

**Table 1:** Prevalence of mental health disorders

Study	Location	Type of detention	Sample size	Assessment tool	Anxiety disorder (%)	Depressive disorder (%)	PTSD (%)	PRS (%)
Amarasena et al (2023)^44^	Australia	Offshore, held, indefinite	*N* = 62	Clinical interview	–	13	13	15
Ehntholt et al (2018)^33^	UK	Onshore, held, definite	*N* = 35	SCID-IV	–	9	34	–
Mares and Jureidini (2004)^45^	Australia	Remote onshore, held, indefinite	*N* = l0	Clinical interview	70	100	100	–
Médecins Sans Frontières (MSF) (2018)^30^	Australia	Offshore, non-held, indefinite	*N* = 39	Clinical interview	5	44	15	26
Steel et al (2004)^47^	Australia	Remote onshore, held, indefinite	*N* = 20	K-SADS-PL	50	95	50	
Tosif et al (2023)^31^	Australia	Offshore/onshore, held, indefinite	*N* = 277	Clinical interview	44	32	30	

PTSD, post-traumatic stress disorder; PRS, pervasive refusal syndrome.

The prevalence of major depressive disorder (MDD) ranged from 9% to 100%, anxiety disorder from 5% to 70%, PTSD from 13% to 100% and PRS from 15% to 26%. Tosif et al^31^ found the prevalence of mental health diagnoses was significantly higher (all *p* = 0.001) in children held in offshore detention on Nauru, compared with those who experienced Australian detention (any mental health disorder 82% versus 54%; depression 54% versus 24%; anxiety 63% versus 36%; and PTSD 49% versus 22%).

Meta-analyses were completed for PTSD (*k* = 7) and MDD (*k* = 7) from six studies. The two independent samples reported within Tosif et al^31^ were included as separate effect sizes. For PTSD, the mean diagnosis prevalence was 32.0% [95% CI 19.4, 48.0]. The *Q* value was 41.49 (*df* = 6, *p* < 0.001), indicating heterogeneity across the samples. For MDD, the mean diagnosis prevalence was 42.2% [95% CI 22.9, 64.3]. The *Q* value was 65.93 (*df* = 6, *p* < 0.001), indicating heterogeneity across the samples. Although the variability across effect sizes was significant for both diagnoses, a high proportion of the variability reflected real differences, as indicated by *I* ^2^ (PTSD = 86%, MDD = 91%). Supplementary Figs 1 and 2 show the forest plots for these analyses. Meta-analyses were not conducted for anxiety disorders (*k* = 4), self-harm and suicidality (*k* = 5) or PRS (*k* = 2) because of the small number of studies, likely resulting in unreliable findings.

### Quality appraisal of studies

Supplementary Table 6 provides a quality appraisal of the 21 included studies. Several studies had small sample sizes with limited data on non-responders, many studies relied upon convenience sampling (e.g. recruitment through offers of legal assistance or assessing children attending mental health services), which may have biased the selection of participants, and not all studies used standardised instruments. Despite these limitations, the quality ratings of the included papers were generally high.

## Discussion

This systematic review identifies a high burden of mental health symptoms in children who have experienced immigration detention. The results consistently demonstrate that immigration detention is associated with severe adverse effects on the mental health of children across multiple reception countries (e.g. the UK, Australia, the USA, Denmark, Finland, the Netherlands, Libya), forms of detention used and regardless of duration. There is however, evidence of a dose effect, with the prevalence of mental distress being most pronounced in children in held, indefinite protracted detention (e.g. Australian immigration detention centres). Mental health difficulties were still evident in children in other types of detention such as held, definite, short detention (e.g. USA immigration detention centres) and non-held, definite, prolonged detention (e.g. European reception centres). Saliently, even short periods of detention (e.g. <1 month) and prolonged stays in open reception centres are associated with significant harm to children’s mental health. Similarly high rates of post-traumatic stress were reported in two studies of unaccompanied minors, a group that is likely to be particularly vulnerable to poor mental health.

Common symptoms of mental distress included self-harm, suicidal ideation, emotional problems, low mood, anxiety, post-traumatic stress and sleep difficulties. Physical symptoms that were frequently observed included headaches and enuresis. Fewer studies reported on children’s development, although those that did identified significant parent concerns about children’s developmental progress. Fewer studies reported on abnormal attachment issues, an area requiring further investigation. The most frequently reported mental health disorders were MDD, PTSD, anxiety disorders and PRS. Our meta-analysis found the prevalence of PTSD was 32.0% [95% CI 19.4, 48.0] and that of MDD was 42.2% [95% CI 22.9, 64.3] across the detention samples with a high degree of heterogeneity across studies, as expected given variability in conditions. Children’s detention experience was typically accompanied by exposure to violence, concurrent parental and/or sibling mental illness, family separations, disrupted schooling and forced relocations between detention centres.

This systematic review has several notable strengths. It is the first systematic review to focus on the impact of a broad range of detention experiences on children’s mental health. The use of broad inclusion criteria has enabled analysis of studies reporting on children in different detention settings, including open European reception centres.^38,40^ This is important because it allows a comparison between types of detention (e.g. prolonged/short, held/non-held, indefinite/definite) and prevalence of mental health concerns. This review is also the first to include data from the grey literature, notably the MSF report^30^ – one of only six studies reporting on mental health diagnoses. Other strengths are adherence to the PRISMA guidelines, use of a comprehensive search strategy and independent screening, data extraction and quality appraisal of studies. It is unlikely we have missed published data, noting that government data are not publicly available.

As previous reviews have noted, methodological features may affect generalisability of the reported findings. Cross-sectional study designs make it difficult to isolate the impact of the detention experience, also considering children’s potential exposure to other traumatic events during pre- and peri-migratory phases.^4,11,20^ Regardless, there remains considerable evidence suggesting that the experience of detention has an independent, detrimental effect upon children’s mental well-being. Steel et al^47^ retrospectively assessed mental health diagnoses before detention and found that children had a tenfold increase in mental health morbidity after entering detention. Reijneveld et al^42^ examined outcomes for unaccompanied minors in held detention compared with non-held detention (with greater autonomy) and found that held detention was associated with greater emotional problems. Zwi et al^28^ compared SDQ data in detained and non-detained asylum-seeker children, finding that children living in detention had more clinically significant emotional and behavioural difficulties compared with children living in the community. Research in adult populations^23,24^ has also suggested detention experiences may exacerbate existing difficulties and/or contribute to/cause new mental health problems. Despite the cross-sectional nature of most studies, the best available data suggest detention experience contributes to the high prevalence of mental distress identified.

Generalisability of findings is affected by small sample sizes and use of convenience sampling. In six studies reporting on mental health diagnoses, two reported on small numbers of children (Mares and Jureidini,^45^ *n* = 10; MSF,^30^ *n* = 39) accessing mental health services, one reported on a small group of adolescents seeking compensation for illegal detention (Ehntholt et al,^33^ *n* = 35) and another on a small number of children from families seeking legal assistance (Steel et al,^47^ *n* = 20). Other studies have relied on recruitment in community areas within detention centres. This may have biased recruitment towards higher functioning detainees who were more likely to be socially engaged.^35^ Conversely, studies recruiting participants requiring legal assistance for their asylum applications^34,47^ may be biased towards including more vulnerable children and families. Thus, the use of convenience sampling may result in either an underestimation or overestimation of mental distress. Hanes et al^12^ reported that almost half (43%) of the children seeking asylum had evidence of acute severe psychological distress, with the strength of the study being statewide capture of patients through a centralised service with many experiencing detention across Australian sites (mainland and offshore).

It should also be noted that there was limited reporting of validated, standardised assessment measures, with the potential for either under- or overestimation of diagnosis. Many studies relied on clinical interviews to assess mental health symptoms and diagnoses, making it difficult to determine the quality or rigour of assessments. Amarasena et al^44^ reported that anxiety, low mood, self-harm and suicidal ideation were frequent (36–47%), but only diagnosed MDD in 13%, suggesting underdiagnosis. However, many clinical assessments were completed by paediatricians experienced in best practice trauma-informed care and refugee health. It is also important to consider the limitations of DSM-5 oriented assessment measures (e.g. SCID-IV, K-SADS-PL, PTSDRI), which may lack validity evidence for use in this population, or with interpreters, and may not adequately capture cultural idioms of distress, especially considering the diverse range of cultural and language backgrounds in asylum-seeker children. Concerns around SDQ utility in refugee children compared to comprehensive multidisciplinary clinical assessment have been reported previously.^17^

Somatic complaints, such as increased body heat, sleep paralysis and panic symptoms, compared with avoidance reactions, are more commonly reported in non-Western cultures.^50,51^ Children’s cumulative trauma exposure may also result in symptom clusters that are not well described by the DSM-5 or brief self-report measures such as the SDQ.^4^ For instance, the DSM-5 does not recognise PRS (identified in two included studies),^30,44^ which is characterised by apathy and profound withdrawal, including refusal to eat, drink, talk and walk, and has been described in asylum-seeker children in detention.^52,53^ These methodological issues are largely attributable to the practical and ethical challenges of conducting research within a highly politicised environment, where governments have historically made it very difficult for independent oversight of the well-being of people seeking asylum.

Finally, our systematic review was limited to English language articles and identifies detention regimens operated in and by high-income countries (Fig. 2), and therefore will not provide a complete international picture of the impact of immigration detention on children’s mental health. There was a predominance of studies from Australia, which may limit the generalisability of the findings to the global immigration detention population. Large-scale migration-related detention of children has been documented in many other countries, including Indonesia, Israel, Thailand, Malaysia and Mexico,^54,55^ although to our knowledge no research to date has examined the mental health of asylum-seeking children in these settings. For example, Human Rights Watch^54^ has reported that children detained in Thailand are held in dirty cells and deprived of food, education and the opportunity to exercise. The prevalence of mental health disorders may be even higher for children detained in these conditions.

This review synthesises the findings of 21 studies, reporting on 9620 children held in immigration detention across eight countries, providing the highest quality current evidence on the impact of immigration detention on children’s mental health. The findings demonstrate that children in immigration detention experience an extremely high prevalence of mental distress across a range of detention settings. The analyses are consistent with the view that immigration detention harms children, and any period of being held in a detention setting is associated with poor mental health outcomes. Offshore, prolonged, held detention settings are profoundly harmful for children and have increased adverse impact on mental health.

Our findings point to the urgent need to develop trauma-informed treatment approaches for children and families who have experienced detention, and to ensure these are culturally appropriate, accessible and implemented using child and family-centred approaches. More importantly, destination countries such as Australia cannot justify continued implementation of immigration detention for children and families, and other countries such as the UK and USA should not introduce held immigration detention or base policy on models shown to be harmful. It is vital that governments implement humane immigration policies that uphold basic human rights and end the practice of detaining children and families seeking asylum or refuge.

## Supporting information

Priestley et al. supplementary material Priestley et al. supplementary material

## Data Availability

As a systematic review, this study used data already reported in the primary literature or in grey literature in the public domain. Summary data supporting the findings of this study are available within the article and supplementary materials.

## Supplementary material

The supplementary material can be found at https://doi.org/10.1192/bjp.2025.29
