# Supplementary material for: The impact of immigration detention on children’s mental health: systematic review
Source: Br J Psychiatry. 2025 Apr 10;227(6):870–9. doi: 10.1192/bjp.2025.29 (PMC12628124; doi:10.1192/bjp.2025.29)
Supplement: Priestley et al. supplementary material [file S0007125025000297sup001.docx]

**Supplementary Table 1**

*Search Strategy*

| **PsycINFO:** |
| --- |
| 1. exp Mental Disorders/ 2. exp Mental Health/ 3. exp Major Depression/ 4. exp Psychiatric Symptoms/ 5. exp “Depression (Emotion)”/ 6. exp Anxiety Disorders/ or exp Anxiety/ 7. exp Trauma/ or exp “Stress and Trauma Related Disorders”/ 8. exp Attempted Suicide/ or exp Suicide/ 9. exp Behavior Problems/ 10. exp Well Being/ 11. exp Somatoform Disorders/ 12. exp Psychodiagnosis/ 13. exp Psychological Stress/ 14. exp Personality Disorders/ 15. exp Conduct Disorder/ 16. 1 or 2 or 3 or 4 or 5 or 6 or 7 or 8 or 9 or 10 or 11 or 12 or 13 or 14 or 15 17. exp Immigration/ 18. exp Legal Detention/ 19. exp Correctional Institutions/ 20. exp Incarceration/ 21. detention center/ or detention centre/ or detention camp/ or detention basin/ 22. offshore processing centre*.mp. 23. asylum seeker center/ or asylum centre.mp. 24. immigration detention*.mp. 25. (depriv* adj2 liberty).mp. 26. (detain or detained).mp. 27. imprison*.mp. 28. incarcerat*.mp. 29. (reception adj cent*).mp. 30. (asylum adj1 cent*).mp. 31. (accommodation adj1 cent*).mp. 32. temporary protection.mp. 33. custod*.mp. 34. 17 or 18 or 19 or 20 or 21 or 22 or 23 or 24 or 25 or 26 or 27 or 28 or 29 or 30 or 31   or 32 or 33   1. exp Pediatrics 2. exp Adolescent Psychology/ or exp Adolescent Behavior/ or exp Adolescent Health/ or exp Adolescent Development/ or exp Adolescent Psychiatry/ or exp Adolescent Psychopathology/ or exp Adolescent Psychotherapy/ 3. exp Child Behavior/ or exp Child Psychiatry/ or exp Child Psychotherapy/ or exp Child Psychology/ or exp Child Health/ or exp Child Psychopathology/ 4. unaccompanied minor*.mp. 5. unaccompanied migrant youth*.mp. 6. unaccompanied migrant child*.mp. 7. migrant children*.mp. 8. 35 or 36 or 37 or 38 or 39 or 40 or 41 9. exp Refugees/ 10. exp Asylum Seeking/ 11. exp Political Asylum/ 12. exp Human Migration/ 13. (asylumseeker* or asylum-seeker*).mp. 14. asylum applicant*.mp. 15. (asylum adj1 claim*).mp. 16. (refuge* or migrant* or immigrant*).mp. 17. immigration detainee*.mp. 18. 43 or 44 or 45 or 46 or 47 or 48 or 49 or 50 or 51 19. 16 and 34 and 42 and 52 |
| **Embase:** |
| 1. mental health/ 2. emotional disorder/ 3. conduct disorder/ 4. anxiety disorder/ 5. depression/ 6. mental disease/ 7. posttraumatic stress disorder/ 8. personality disorder/ 9. suicide/ 10. mental stress/ 11. developmental disorder/ 12. psychological well-being/ 13. melancholia/ 14. distress syndrome/ 15. psychiatric diagnosis/ 16. psychosis/ 17. somatoform disorder/ 18. anxiety/ 19. suicide attempt/ 20. suicidal ideation/ 21. psychosocial disorder/ 22. psychotrauma/ 23. 1 or 2 or 3 or 4 or 5 or 6 or 7 or 8 or 9 or 10 or 11 or 12 or 13 or 14 or 15 or 16 or 17   or 18 or 19 or 20 or 21 or 22   1. adolescent/ 2. child/ 3. preschool child/ 4. school child/ 5. child parent relation/ 6. infant 7. unaccompanied minor*.mp. 8. unaccompanied migrant youth*.mp. 9. unaccompanied migrant children*.mp. 10. migrant children*.mp. 11. young adult/ 12. 24 or 25 or 26 or 27 or 28 or 29 or 30 or 31 or 32 or 33 or 34 13. asylum seeker/ 14. refugee/ 15. prisoner/ 16. forced migrant/ 17. immigrant/ 18. undocumented immigrant/ 19. immigration 20. migration/ 21. (asylumseeker* or asylum-seeker*).mp. 22. asylum applicant*.mp. 23. (asylum adj1 claim*).mp. 24. refuge* or migrant* or immigrant*).mp. 25. immigration detainee*.mp. 26. 36 or 37 or 38 or 39 or 40 or 41 or 42 or 43 or 44 or 45 or 46 or 47 or 48 27. (depriv* adj1 liberty).mp. 28. (detain or detained).mp. 29. imprison*.mp. 30. incarcerat*.mp. 31. (reception adj cent*).mp. 32. (asylum adj1 cent*).mp. 33. (accommodation adj1 cent*).mp. 34. temporary protection.mp. 35. custod*.mp. 36. detention/ 37. immigration detention/ 38. asylum seeker center/ 39. correctional facility/ 40. detention center/ or detention camp/ or detention basin/ 41. legal detention*.mp. 42. offshore processing centre*.mp. 43. 50 or 51 or 52 or 53 or 54 or 55 or 56 or 57 or 58 or 59 or 60 or 61 or 62 or 63 or 64   or 65   1. 23 and 35 and 49 and 66 |
| **MEDLINE:** |
| 1. exp Anxiety/ or exp Anxiety Disorders/ 2. exp Depression/ 3. Mental Disorders/ 4. Mental Health/ 5. Child Behavior Disorders/ or Problem Behavior 6. Stress Disorders, Post-Traumatic/ 7. Stress, Psychological/ 8. Self-Injurious Behavior/ 9. Suicide/ 10. 1 or 2 or 3 or 4 or 5 or 6 or 7 or 8 or 9 11. correctional facilities/ or jails/ or prisons/ 12. Detention.mp. 13. (Depriv* adj2 liberty).mp. 14. (Detain or Detained).mp. 15. Imprison*.mp. 16. Incarcerat*.mp. 17. (Reception adj1 cent*).mp. 18. (Asylum adj1 cent*).mp. 19. (Accommodation adj1 cent*).mp. 20. Temporary protection.mp. 21. Custod*.mp. 22. Immigration detention*.mp. 23. 11 or 12 or 13 or 14 or 15 or 16 or 17 or 18 or 19 or 20 or 21 or 22 24. (asylum adj1 seek*).mp. 25. (asylumseeker* or asylum-seeker*).mp. 26. Asylum applicant*.mp. 27. (Asylum adj1 claim*).mp. 28. (Refuge* or Migrant* or Immigrant*).mp. 29. Refugees.mp. 30. Unaccompanied minor*.mp. 31. Unaccompanied migrant youth*.mp. 32. Unaccompanied migrant children*.mp. 33. Immigration detainee*.mp. 34. Undocumented migrant*.mp. 35. “emigrants and immigrants”/ or “transients and migrants”/ 36. “Emigration and Immigration”/ 37. 24 or 25 or 26 or 27 or 28 or 29 or 30 or 31 or 32 or 33 or 34 or 35 or 36 38. Adolescent/ 39. Child/ 40. Child, Preschool/ 41. Young Adult/ 42. Infant/ 43. 38 or 39 or 40 or 41 or 42 44. 10 and 23 and 37 and 43 |

**Supplementary Table 2**

*Detention Type/Severity and Characteristics of Children’s Reported Experience in Detention*

| **Author & Year** | **Detention Type** | **Time Spent in Detention** | **Children witnessing violence in detention** | **Parental mental health** | **Family separation** | **Disruption to schooling** | **Relocations** |
| --- | --- | --- | --- | --- | --- | --- | --- |
| Amarasena et al. (2023) | Offshore detention centre (Nauru), held, indefinite | Range 0-4 years  ≥4 years (52%)  Protracted | Not reported | Y (73% parents had mental health diagnosis/severe mental health concern) | Y (18% children separated from primary relatives in detention) | Y (77% experienced disruption to schooling) | Not reported |
| Tosif et al. (2023)  [1-Offshore sample] | Offshore detention centre (Manus Island or Nauru), held/non-held, indefinite | Median 51 months (IQR 29-60)  Protracted | Y (33% witnessed physical violence, 25% witnessed people self-harm) | Y (86% parents reported mental health problems) | Y (21% children separated from at least one parent during detention for weeks-months) | Y (46% children experienced disruptions to schooling) | Y (Children detained in multiple centres; Median 3, Range 1-8) |
| Elliot & Gunasekera (2016) | Onshore detention centre (Wickham Point, Darwin), held, indefinite | Range 3-18 months  Mean 9 months (all children previously held on Nauru for additional 3-17 months)  Prolonged | Not reported | Not reported | Not reported | Y (Many children reportedly unable to concentrate due to PTSD, experiences of bullying because they were from a detention centre) | Not reported |
| Mares (2016) | Offshore detention centre (Christmas Island), held, indefinite | Range 90-390 days  Mean 209.5 days (7 months; SD=62.36 days)  Prolonged | Y (Children exposed to adult violence and self-harm) | Y (83.2% parents were likely to have severe mental disorder) | Y (38% children were unaccompanied minors. Accompanied children also separated from family separations.) | Y (Children received only a few weeks of schooling) | Not reported |
| Hanes et al. (2019)  [1 - Offshore sample] | Offshore detention centre (Christmas Island), held, indefinite | Median 7 months (IQR 3-12.5)  Prolonged | Y (100% had witnessed trauma) | Y (53.6% parents had severe mental health issues) | Y (84% had experienced family separation, 47% experienced ongoing separation) | Y (89% experienced disruption to schooling, only 43% had access to school whilst in detention) | Y (Median 2,  IQR 1-3) |
| Rothe et al. (2002a) | Offshore detention (Guantanamo Camp), held, indefinite | Range 4-6 months  Prolonged | Y (Adolescents witnessed riots, violence, suicide attempts) | Not reported | Y (Unaccompanied minors included in sample) | Y (Adolescents refused to attend school, preferred to remain with family) | Not reported |
| Rothe et al. (2002b) | Offshore detention (Guantanamo Camp), held, indefinite | Range 6-8 months  Prolonged | Y (80% adolescents witnessed violence, 37% witnessed suicide attempt/s) | Not reported | Y (19% separated from family in detention). | Not reported | Not reported |
| MSF (2018) | Offshore detention centre (Nauru), non-held, indefinite | Not reported | Y (reports of asylum seekers witnessing traumatic events on Nauru including physical and sexual violence) | Not reported | Y (13% total sample, including adults, separated from family in detention) | Not reported | Not reported |
| Steel et al. (2004) | Remote onshore detention centre, held, indefinite | Range 2 years – 2 years 8 months  Mean 2 years 2 months  Protracted | Y (100% witnessed people self-harm or attempt suicide, 95% witnessed physical assault) | Y (100% parents met criteria for mental health disorder/s) | Y (42% forcibly separated from family in detention e.g., parent placed in solitary confinement) | Y (42% reported poor access to schooling) | Y (Families had been held in multiple detention centres since arrival in Australia) |
| Mares & Jureidini (2004) | Remote onshore detention centre, held, indefinite | Range 12-18 months  Mean 15 months  Protracted | Y (100% children had witnessed hangings, slashings, and self-poisoning) | Y (100% children had at least one parent affected by mental illness, 87% parents met criteria for mental health disorder) | Not reported | Y (Children reported anxiety about falling behind in schoolwork) | Not reported |
| Tosif et al. (2023)  [2 -Onshore sample] | Onshore detention centre (Melbourne) , held, indefinite | Median 7 months (IQR 4-16 months)  Prolonged | Y (33% witnessed physical violence) | Y (41% of parents had mental health issues) | Y (21% separated from at least one parent for weeks-months) | Y (46% children experienced disruption. Many children not enrolled in school for months). | Y (Children detained in multiple centres, median 3, range 1-8) |
| Hanes et al. (2019)  [2 -Onshore sample] | Onshore detention centre (Perth), held, indefinite | Median 7 months (IQR 3-12.5)  Prolonged | Y (100% had witnessed trauma) | Y (53.6% parents had severe mental health issues) | Y (84% had experienced family separation, 47% experienced ongoing separation) | Y (89% experienced disruption to schooling, only 43% had access to school whilst in detention) | Y (Median 2  IQR 1-3) |
| Lorek et al. (2009) | Onshore detention centre (Yarl’s Wood), held, indefinite | Range 11-155 days  Median 43 days  Prolonged | Not reported | Not reported | Y (at least 12 children separated from primary caregiver in detention) | Y (children said they were not learning anything, missed having a ‘real school’) | Not reported |
| Ehntholt et al. (2018) | Onshore detention centre, held, indefinite | Mean 22.8 days (SD=21)  Range 4-92 days  Short | Y (18% pushed or touched aggressively by officer, 27% witnessed fights between officers and other detainees, 51% witnessed fights between detainees) | N (sample comprised of unaccompanied minors) | Y (100% unaccompanied minors. 87% unwantedly separated from family) | Y (51% had no access to English lessons or any schooling) | Y (30% children suddenly moved to another detention centre) |
| Young & Gordon (2016) | Onshore detention centre, held, indefinite | Range 10-30 days  (assessed within 10-30 days of arrival)  Short | Not reported | Not reported | Not reported | Not reported | Not reported |
| Derluyn et al. (2023) | Onshore detention centre, held, indefinite | Not reported | Y (71% experienced physical violence, 74% witnessed physical violence) | N (sample comprised of unaccompanied minors) | Y (100% unaccompanied minors. 5% forcibly separated from family since arrival in Libya) | Y (70.5% children reported not receiving enough education) | Not reported |
| Reijneveld et al. (2005)  [1-Held sample] | Onshore campus, held, definite | Range 0-6 months  Prolonged | Not reported | N (sample comprised of unaccompanied minors) | Y (100% unaccompanied minors) | Y (education program emphasised repatriation to country of origin, offered no opportunities to learn Dutch language) | Not reported |
| MacLean et al. (2019) | Onshore detention centre, held, definite | Range 1-44 days  Mean 9 days (SD=6)  Short | Not reported | Not reported | Y (17% previously separated from mothers in detention) | Not reported | Y (17% previously transferred from other detention centres) |
| Nielsen et al. (2008) | Onshore asylum centre, non-held, indefinite | Range 1-91 months, Mean 48.4 months  Protracted | Not reported | Not reported | Not reported | N (All school age children attended asylum centre school or municipal or private school) | Y (81% children/families relocated 4-13 times, Mean 5.6, Range 0-13) |
| Hanes et al. (2019) [3 - Community detention sample] | Onshore community detention, non-held, indefinite | Median 7 months (IQR 3-12.5)  Prolonged | Y (100% had witnessed trauma) | Y (53.6% parents had severe mental health issues) | Y (84% had experienced family separation, 47% experienced ongoing separation) | Y (89% experienced disruption to schooling, only 43% had access to school whilst in detention) | Y (Median 2  IQR 1-3) |
| Jakobsen et al. (2017) | Onshore reception centre, non-held, indefinite | Range 3 weeks – 2 years 2 months (Assessed at 3 weeks)  Short | Not reported | N (sample comprised of unaccompanied minors) | Y (100% unaccompanied minors) | Y (Minors placed in adult reception centre were unable to attend school) | Not reported |
| Sourander (2003) | Onshore asylum centre, non-held, definite | Range 2-22 months  Mean 7 months  Prolonged | Not reported | Y (69% parents reported mental health issues, 19% reported suicide attempt) | Y (60% separated from families including death or disappearance of family member) | Not reported | Not reported |
| Sourander (1998) | Onshore asylum centre, non-held, definite | Range 4-6 months  Mean 5 months  Prolonged | Not reported | N (sample comprised of unaccompanied minors) | Y (100% unaccompanied minors) | Not reported | Not reported |
| Reijneveld et al. (2005)  [2-Non-held sample] | Onshore reception centre, non-held, definite | Range 0-6 months  Prolonged | Not reported | N (sample comprised of unaccompanied minors) | Y (100% unaccompanied minors) | Y (Education if available was primarily aimed at learning Dutch language) | Not reported |
| Eiset et al. (2020) | Onshore reception centre, non-held, definite | Range 0-10 days  Short | Not reported | Not reported | Y (86% separated from family members during refugee journey) | Not reported | Not reported |


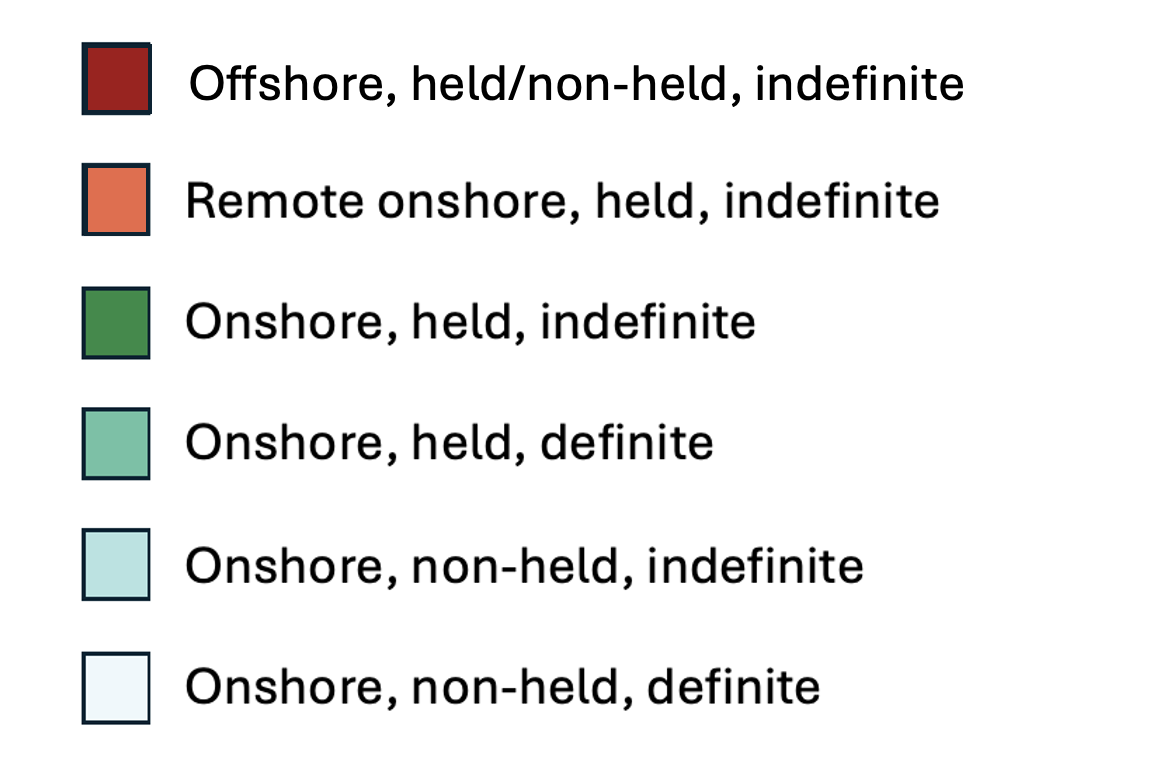


**Supplementary Table 3**

*Characteristics of Included Studies*

| **Author & Year** | **Study Design & Sample** | **N** | **Female %** | **Age** | **Year/s of Assessment** | **Country of Origin** | **Reception**  **Country** | **Method of Assessment** |
| --- | --- | --- | --- | --- | --- | --- | --- | --- |
| Amarasena et al. (2023) | Cross-sectional. Children and young people assessed around time of transfer from offshore detention. Recruited from ten health sites. | 62 | 39 | Range 3 months – 22 years  Mean=9 years (SD=5.7) | 2013-2019 | Top 3 countries: Iran (47%), Iraq (11%), Sri Lanka (10%) | Australia | Clinical interview and survey administered by specialist clinician |
| Derluyn et al. (2023) | Cross-sectional. Unaccompanied minors recruited via snowball sampling from relevant NGOs. | 99 | 5.1 | Not reported | 2018  Apr-Jul | Eritrea (50.5%), Somalia (26.3%), Nigeria (5%), Sudan (5%), %), Cote D'Ivoire (2%), Mali (2%), Sierra Leone (2%), Chad (1%), Ethiopia (1%), Liberia (1%), Senegal (1%) | Libya | Clinical interview, RATS, HSCL-37 |
| Ehntholt et al. (2018) | Cross-sectional. Unaccompanied minors subject to age dispute procedures seeking compensation for illegal detention. Previously held within British detention centres and assessed 3 years post-detention. | 35 | 40 | During detention Range=13-17 years; Mean=15.8 years (SD=0.93)  At assessment  Range=16-21 years Mean=19.1 years (SD=1.12) | 2008-2009 (approx. 3 years after detention)  Interval from detention to assessment: Range 18-74 months, Mean 37.5 months (SD=11 months) | Afghanistan (31%), DRC (9%), Iran (9%), Uganda (9%), China (6%), Ivory Coast (6%), Eritrea (3%), India (3%), Kosovo (3%), Malawi (3%), Mongolia (3%), Morocco (3%), Nigeria (3%), Sierra Leone (3%), Somalia (3%), Sri Lanka (3%), Vietnam (3%) | ~~England~~  UK | Clinical interview, SCID-IV for PTSD and MDD, Detention Experiences Checklist – UK version, Stressful Life Events Questionnaire, RATS |
| Eiset et al. (2020) | Cross-sectional. Asylum-seeking minors offered a voluntary health assessment within 10 days arrival. | 7210 | 43 | Range 0-17 years | 2011-2015 | Syria (38%), Russia (12%), Stateless (9.6%), Former Yugoslavia (8.6%), Afghanistan (7.1%), Iran (4.8%), Somalia (3.9%), Eritrea (2.9%), Iraq (2.6%) | Denmark | Clinical interview performed by a nurse experienced in child health |
| Elliott & Gunasekera (2015) | Cross-sectional. Children and parents held in detention. Data collected in monitoring visit by AHRC. | 127 | Not reported | 0-17 years | 2015  Single visit Oct | Ethnic/language groups: Arabic; Bengali; Burmese/Myanmar; Farsi/Persian; Indonesian; Mandarin; Nepali; Rohingyan; Somali; Tamil and Vietnamese | Australia | CTSQ (Self-report), PEDS, HOPES |
| Hanes et al. (2019) | Retrospective audit medical records. Children attending paediatric refugee health service. | 110 | 44.5 | Mean=6 years (SD=4.7) | 2012- 2016 | Iran (35.5%), Sri Lanka (21.8%), Iraq (15.5%), Afghanistan (10%), Burma (9.1%), Other (8.82%) | Australia | Standardised refugee health screening completed by clinic staff |
| Jakobsen et al. (2017) | Longitudinal cohort study. Unaccompanied asylum seeker minors (male). Convenience sampling used to recruit recent arrivals. | 138 | 0 | Self-reported age; Range 14-20 years, Mean16.2 years (SD=0.84)  Age assessed by authorities; Range15-27 years, Mean 18.22 years (SD=2.27) | 2009 –2011 | Afghanistan (73.9%), Somalia (23.2%), Iran (2.2%), Algeria (0.7%), Other | Norway | HSCL-25 (Self-report), HTQ (Self-report) |
| Lorek et al. (2009) | Cross-sectional. Children and families seeking asylum requesting legal assistance. | 24 | 50 | Range 3 months-17 years, Mean 4.75 years | 2006  Feb-Aug | Nigeria (25%), Uganda (21%), Pakistan (13%), Jamaica (13%), Congo-Brazzaville (8%), DRC (8%), Central African Republic (8.3%), Ghana (4%) | ~~England~~  UK | Clinical interview conducted by psychologist/paediatrician; SCAS (self-report), SDQ (Parent-report), DSRS (Self-report) |
| MacLean et al. (2019) | Cross-sectional. Children held at a US immigration detention centre. Convenience sampling used to interview mothers who presented to a visitation centre. | 425 | Not reported | Range 4-17 years  Mean 10 years (SD=4) | 2018 | Honduras (50%), Guatemala (22%), El Salvador (2%), Mexico (2%), Nicaragua (2%), Cuba (1%), Venezuela/Peru/Nigeria/Brazil (1%) | USA | SDQ (Parent-report), PTSDRI (Self-report) |
| Mares (2016) | Secondary analysis of an AHRC dataset. Asylum seeker parents and children. | 191 | Not reported | Range 0-17 years  Mean 7.64 years | 2014  Mar | Iran, Sri Lanka, Iraq, Afghanistan, Vietnam, Somalia, Syria, Stateless (Rohingya) | Australia | K-10 (Self-report), SDQ (Parent-report), Parent-reported concerns |
| Mares & Jureidini (2004) | Cross-sectional. Children held in immigration detention. Consecutive referrals to a CAMHS. | 20 | 30 | Range 11 months-17 years | 2002  Feb-Aug | Iran, Iraq, Afghanistan, Palestine | Australia | Clinical interviews conducted by allied health clinicians and child psychiatrists |
| Médecins Sans Frontières (2018) | Descriptive analysis of patient database. Asylum-seeker parents and children engaged in mental health consultation. | 39 | Not reported | Range 0-17 years | 2017- 2018  Nov-Oct | Iran, Somalia, Myanmar, Afghanistan, India, Lebanon, Sri Lanka, Bangladesh, Iraq | Australia | Clinical interview |
| Nielsen et al. (2008) | Cross-sectional. 95% of parent-accompanied asylum-seeking children at asylum centre. Sampling method not reported. | 246 | 42 | Range 4-16 years | 2006  Oct-Dec | Former Yugoslavia (48%), Iraq (27%), Somalia (4.8%), Afghanistan (0.04%), Azerbaijan (0.04%), Iran (4.5%), Kazakhstan (0.04%), Libya (0.04%), Lithuania (0.04%), Pakistan (0.08%), Russia (3.3%), Sri Lanka (0.04%), Palestine (3.7%), Stateless (2.0%), Syria (0.08%), Ukraine (0.04%) | Denmark | SDQ (Teacher-report) |
| Reijneveld et al. (2005) | Comparative cross-sectional. Unaccompanied adolescent asylum seekers. Sampling method not reported. | 69  (Held detention) | 45 | Range 15-17 years  Mean 16.1 years (SD=0.7) | 2003  Mar-Jul | Africa (87%; Angola 13%, Sierra Leone 13%), Asia (7.2%), Europe (5.8%) | Netherlands | HSCL-25 (Self-report), RATS (Self-report) |
|  |  | 53  (Non-held detention) | 17 | Range 14-18 years  Mean 16.4 years (SD=1.1) | 2003  Mar-Jul | Africa (88.7%; Angola 32%, Sierra Leone 15%), Asia (7.5%), Europe (3.8%) |  |  |
| Rothe et al. (2002a) | Cross-sectional. Children and young people presenting to a psychiatric clinic in a detention centre. | 74 | 36 | Range13-19 years  Mean 15.5 years | 1994-1995  Dec-Mar | Not reported | USA | PTSDRI (Self-report), Symptom checklist on psychological stress (Self-report) |
| Rothe et al. (2002b) | Children and young people previously held in detention who had no premorbid psychiatric conditions whilst detained. Children were sampled from transitional summer schools 4-6 months post-detention. | 87 | 43 | Range 6-17 years  Mean 14.9 years | 1995 | Not reported | USA | PTSDRI (Self-report), CBCL (Teacher report), Questionnaire on exposure to traumatic events (Self-report) |
| Sourander (1998) | Cross-sectional. Unaccompanied refugee minors waiting for placement. Convenience sampling of consecutive arrivals at centre. | 46 | 26 | Range 6-17 years  Mean 14.1 years (SD=2.3) | 1994-1995  Mar-Sep | Somalia (80%), Ethiopia (4.3%), Thailand (4.3%), Vietnam (2.2%), Angola (2.2%), Nigeria (2.2%), Zaire (2.2%), Iraq (2.2%), Burma (2.2%) | Finland | CBCL (completed by centre staff member), Clinical interview |
| Sourander (2003) | Cross-sectional. All children living with their families at an asylum centre. | 25 | Not reported | Range <7-16 years | Late 1990s | Kosovo (44%), Iraq (40%), Afghanistan (16%) | Finland | Clinical interview |
| Steel et al. (2004) | Cross-sectional. Asylum seeker families and children identified by legal professionals helping them challenge legality of detention. | 20 | 41 | Range 3-19 years | Sept 2002- Feb 2003 | Not reported | Australia | K-SADS-PL (clinician-administered), Symptom checklist |
| Tosif et al. (2023) | Retrospective audit of medical records. Asylum seeker children and adolescents attending a paediatric refugee health service. | 79  (Offshore detention) | 47 | Median 1.9 years  (IQR 0-7.0) | 2012- 2021 | Iran (47%), Malaysia (8%), Iraq (5%), Afghanistan (3%), Sri Lanka (X%), India (4%), Lebanon (2%), Yemen (1%), Thailand (1%), Syria (1%), Pakistan (1%), Nepal (1%), Myanmar (1%), Cyprus (1%), Bangladesh (0.03%) | Australia | Clinical interview completed by paediatricians |
|  |  | 198  (Mainland detention) | 48 | Median 4.8 years  (IQR 1.6-8.2) |  |  |  |  |
| Young & Gordon (2016) | Secondary analysis of an AHRC dataset. Children held in immigration detention on mainland Australia. Data collected in routine mental health screening. | 243 | 42 | Range 5-17 years | 2014  Feb-Jun | Not reported | Australia | HoNOSCA (Clinician rated) |

*Note.* Abbreviations: *AHRC* Australian Human Rights Commission, *CAHMS* Child and Adolescent Mental Health Service, *CBCL* Child Behaviour Checklist, *CTSQ* Child Trauma Screening Questionnaire, *DSRS* Birleson Depression Rating Scale, *IQR* Interquarrtile range, *HoNOSCA* Health of Nation Outcomes Scale for Children and Adolescents, *HSCL-25* Hopkins Symptom Checklist 25, *HSCL-37* Hopkins Symptom Checklist 37, *HTQ* Harvard Trauma Questionnaire, *K-SADS-PL* Schedule for Affective Disorders and Schizophrenia for School-Aged Children-Present and Lifetime Version, *K-10* Kessler-10, *PEDS* Parents’ Evaluation of Developmental Status, *PTSDRI* Post-Traumatic Stress Disorder Reaction Index, *RATS* Reaction of Adolescents to Traumatic Stress Inventory, *SCAS* Spence Children’s Anxiety Scale, *SCID-IV* Structured Clinical Interview for DSM-IV, *SD* standard deviation, *SDQ* Strengths and Difficulties Questionnaire, *USA* United States of America.

**Supplementary Table 4**

*Prevalence of Mental Health Symptoms*

|  | **Location** | **Type of Detention** | **Sample Size** | **Anxiety** | **Low Mood** | **PTSD** | **Self-Harm** | **Suicidal Ideation** | **Sleep Difficulties** | **Nightmares** | **Emotional Symptoms (SDQ)** | **Conduct Problems (SDQ)** | **Hyperactivity (SDQ)** | **Peer Problems (SDQ)** | **Total Problems (SDQ)** |
| --- | --- | --- | --- | --- | --- | --- | --- | --- | --- | --- | --- | --- | --- | --- | --- |
| **Author & Year** |  |  |  |  |  |  |  |  |  |  |  |  |  |  |  |
| Amarasena et al. (2023) | Australia | Offshore, held, indefinite | N=62 | 39% | 47% | - | 45%* | 45%* | 47% | - | - | - | - | - | - |
| Ehntholt et al. (2018) | UK | Onshore, held, definite | N=35 | - | - | 80% | - | - | - | - | - | - | - | - | - |
| Eiset et al. (2020) | Denmark | Onshore, non-held, definite | N=7210 | 5% | 2% | - | - | - | 15% | - | - | - | - | - | - |
| Elliot & Gunasekera et al. (2015) | Australia | Onshore, held, indefinite | N=20 | - | - | 95% | - | - | - | - | - | - | - | - | - |
| Jakobsen et al. (2017) | Norway | Onshore, non-held, indefinite | N=133 | - | - | 59% | - | - | - | - | - | - | - | - | - |
| Lorek et al. (2009) | UK | Onshore, held, indefinite | N=24 | 100%  (n=11) | 100%  (n=11) | - | - | - | 91%  (n=11) | - | 64%  (n=11) | 18%  (n=11) | 18%  (n=11) | 55%  (n=11) | 55%  (n=11) |
| MacLean et al. (2019) | USA | Onshore, held, definite | N=425 | - | - | 17% (n=150) | - | - | - | - | 32% | 8% | 8% | 14% | 10% |
| Mares (2016) | Australia | Offshore, held, indefinite | N=70 | - | - | - | - | - | 38%*** | 38%*** | 72% | 36% | 35.7% | 17.1% | 50% |
| Mares & Jureidini (2004) | Australia | Remote onshore, held, indefinite | N=10 | - | - | - | 80% | 100% | 100% | - | - | - | - | - | - |
| Nielsen et al. (2008) | Denmark | Onshore, non-held, indefinite | N=246 | - | - | - | - | - | - | - | 20% | 25% | 27% | 24% | 31% |
| Rothe et al. (2002a) | USA | Offshore, held, indefinite | N=74 | - | - | 95% | - | 20%** | 91% | 85% | - | - | - | - | - |
| Rothe et al. 2002b) | USA | Offshore, held, indefinite | N=87 | - | - | 57% | - | - | - | - | - | - | - | - | - |
| Sourander et al. (1998) | Finland | Onshore, non-held, definite | N=46 | - | - | - | - | 13% | - | - | - | - | - | - | - |
| Steel et al. (2004) | Australia | Remote onshore, held, indefinite | N=20 | - | - | - | 25% | 55% | - | - | - | - | - | - | - |
| Tosif et al. (2023) | Australia | Offshore/Onshore, held, indefinite | N=277 | - | - | - | 10% | - | 43% | 27% | - | - | - | - | - |

*Note. *Self-harm, suicidal ideation & attempts, **Suicidal ideation & attempts. ***Sleep difficulties & nightmares.*

**Supplementary Figure 1**

*Forest Plot Analysis – Prevalence of Major Depressive Disorder (MDD**)*

**Supplementary Figure 2**

*Forest Plot Analysis – Prevalence of PTSD*

**Supplementary Table 5**

*Prevalence of Physical and Developmental Health Symptoms*

| **Author & Year** | **Location** | **Type of Detention** | **Sample Size** | **Headaches** | **Abdominal Pain** | **Enuresis** | **Encopresis** | **Developmental concerns** | **Somatic concerns** | **Language delay/Regression** |
| --- | --- | --- | --- | --- | --- | --- | --- | --- | --- | --- |
| Amarasena et al. (2023) | Australia | Offshore, held, indefinite | 62 | 13% | 16% | - | - | - | - | 6% |
| Eiset et al. (2020) | Denmark | Onshore, non-held, definite | 7210 | 27%* | - | 7% | - | - | - | - |
| Elliot & Gunasekera (2015) | Australia | Onshore, held, indefinite | 34 | - | - | - | - | 50% | - | - |
| Hanes et al. (2019) | Australia | Offshore/onshore, held, indefinite | 109 | - | - | 14% | - | 52%*** | 8% | - |
| Lorek et al. (2009) | UK | Onshore, held, indefinite | 24 | 27% (n=11) | 91% (n=11) | 53% (n=14) | - | 100% (n=8) | - | 50% (n=8) |
| Mares (2016) | Australia | Offshore, held, indefinite | 48 | 8%** | - | 6% | 80% | - | - | 4% |
| Mares & Jureidini (2004) | Australia | Remote onshore, held, indefinite | 10 | - | - | 30% | - | - | 50% | - |
| Rothe et al. (2002a) | USA | Offshore, held, indefinite | 74 | - | - | 47% | 9.5% | - | - | - |
| Sourander (2003) | Finland | Onshore, non-held, definite | 25 | - | - | - | - | 16% | - | - |
| Steel et al. (2004) | Australia | Remote onshore, held, indefinite | 20 | - | - | 20% | - | - | - | - |
| Tosif et al. (2023) | Australia | Offshore/onshore, held, indefinite | 277 | 10% | - | 19% | 5% | 38% | 18% | - |

*Note. *included toothache, **included nail-biting, *** reported on combined cognitive, educational and developmental concerns*

**Supplementary Table 6**

*Quality Appraisal of Included Studies*

| **Author & Year** | **Q1** | **Q2** | **Q3** | **Q4** | **Q5** | **Q6** | **Q7** | **Q8** | **Q9** | **Q10** | **Q11** | **Q12** | **Q13** | **Q14** | **Q15** | **Q16** | **Q17** | **Q18** | **Q19** | **Q20** |
| --- | --- | --- | --- | --- | --- | --- | --- | --- | --- | --- | --- | --- | --- | --- | --- | --- | --- | --- | --- | --- |
| Amaresena et al. (2023) | Yes | Yes | No | Yes | Yes | No | Yes | Yes | No | No | Yes | Yes | Yes | Yes | Yes | Yes | Yes | Yes | No | Yes |
| Derluyn et al. (2023) | Yes | Yes | Yes | Yes | Yes | Yes | No | Yes | Yes | No | Yes | Yes | Yes | No | Yes | Yes | Yes | Yes | No | Yes |
| Ehntholt et al. (2018) | Yes | Yes | No | Yes | Yes | No | No | Yes | Yes | No | Yes | Yes | Yes | No | Yes | Yes | Yes | Yes | No | Yes |
| Eiset et al. (2020) | Yes | Yes | No | Yes | Yes | Yes | No | Yes | No | No | Yes | Yes | Yes | No | Yes | Yes | Yes | Yes | No | Yes |
| Elliott & Gunasekera (2015) | Yes | Yes | No | Yes | Yes | Yes | No | Yes | Yes | No | yes | Yes | No | No | Yes | Yes | Yes | No | No | Yes |
| Hanes et al. (2019) | Yes | Yes | No | Yes | Yes | No | N/A | Yes | No | No | Yes | Yes | No | N/A | Yes | Yes | Yes | Yes | No | Yes |
| Jakobsen et al. (2017) | Yes | Yes | No | Yes | Yes | Yes | No | Yes | Yes | Yes | Yes | Yes | No | No | Yes | Yes | Yes | Yes | No | Yes |
| Lorek et al. (2009) | Yes | Yes | No | Yes | Yes | No | No | Yes | Yes | No | Yes | Yes | Yes | No | Yes | Yes | Yes | Yes | No | Yes |
| MacLean et al. (2019) | Yes | Yes | No | Yes | Yes | No | No | Yes | No | No | Yes | Yes | Yes | No | Yes | Yes | Yes | Yes | No | Yes |
| Mares (2016) | Yes | Yes | No | Yes | Yes | Yes | No | Yes | Yes | No | Yes | Yes | Yes | No | Yes | Yes | Yes | Yes | No | Yes |
| Mares & Jureidini (2004) | Yes | Yes | No | Yes | Yes | No | No | Yes | No | No | Yes | Yes | No | No | Yes | Yes | Yes | Yes | No | Yes |
| Medecins Sans Frontieres (2018) | Yes | Yes | Yes | Yes | Yes | No | No | Yes | No | No | Yes | Yes | No | No | Yes | Yes | Yes | No | No | Yes |
| Nielsen et al (2008) | Yes | Yes | No | yes | Yes | Yes | Yes | Yes | Yes | No | Yes | Yes | No | No | Yes | Yes | Yes | Yes | No | Yes |
| Reijneveld et al. (2005) | Yes | Yes | No | Yes | yes | Yes | No | Yes | Yes | No | Yes | No | Yes | Yes | Yes | Yes | Yes | Yes | No | Yes |
| Rothe et al. (2002a) | No | Yes | No | Yes | Yes | No | No | Yes | yes | No | Yes | No | No | No | Yes | Yes | Yes | Yes | No | No |
| Rothe et al. (2002b) | Yes | Yes | No | Yes | Yes | No | No | Yes | Yes | No | Yes | Yes | No | No | Yes | Yes | Yes | No | No | Yes |
| Sourander (1998) | Yes | Yes | No | Yes | Yes | Yes | No | Yes | Yes | No | Yes | No | No | No | yes | Yes | Yes | Yes | No | No |
| Sourander (2003) | Yes | Yes | No | Yes | Yes | No | N/A | Yes | No | No | Yes | No | No | N/A | Yes | Yes | Yes | Yes | No | Yes |
| Steel et al. (2004) | Yes | Yes | No | Yes | Yes | Yes | No | Yes | Yes | No | Yes | Yes | Yes | No | Yes | Yes | Yes | Yes | No | Yes |
| Tosif et al. (2023) | Yes | Yes | No | Yes | Yes | No | No | Yes | No | Yes | Yes | Yes | Yes | No | Yes | Yes | Yes | Yes | No | Yes |
| Young (2016) | Yes | Yes | No | Yes | Yes | No | N/A | Yes | Yes | No | Yes | No | Yes | No | Yes | Yes | Yes | Yes | No | Yes |

**List of Included Papers**

1. Amarasena L, Samir N, Sealy L, Hu N, Rostami MR, Isaacs D, et al. Offshore detention: cross-sectional analysis of the health of children and young people seeking asylum in Australia. Archives of Disease in Childhood. 2023 Mar;108(3):185–91.
2. Derluyn I, Orsini G, Verhaeghe F, Elhaj R, Lietaert I, Pfeiffer E. The impact of trauma and daily hardships on the mental health of unaccompanied refugee minors detained in Libya. BJPsych Open. 2023 Jan;9(1).
3. Ehntholt KA, Trickey D, Harris Hendriks J, Chambers H, Scott M, Yule W. Mental health of unaccompanied asylum-seeking adolescents previously held in British detention centres. Clinical Child Psychology and Psychiatry. 2018 Mar 22;23(2):238–57.
4. Eiset AH, Loua AS, Kruse A, Norredam M. The health status of newly arrived asylum-seeking minors in Denmark: a nationwide register-based study. International Journal of Public Health. 2020 Oct 21;65(9):1763–72.
5. Elliott E, Gunasekera H. The health and wellbeing of children in immigration detention. Report to the Human Rights Commission Monitoring Visit to Wickham Point Detention Centre, Darwin, NT [Internet]. Australian Human Rights Commission; 2015 [cited 2024 Jul 14]. Available from: https://humanrights.gov.au/our-work/asylumseekers-and-refugees/publications/health-and-well-being-children-immigration
6. Hanes G, Chee J, Mutch R, Cherian S. Paediatric asylum seekers in Western Australia: identification of adversity and complex needs through comprehensive refugee health assessment. Journal of Paediatrics and Child Health. 2019 Nov;55(11):1367-73.
7. Jakobsen M, DeMott MA, Wentzel-Larsen T, Heir T. The impact of the asylum process on mental health: a longitudinal study of unaccompanied refugee minors in Norway. BMJ open. 2017 Jun 1;7(6):e015157.
8. Lorek A, Ehntholt K, Nesbitt A, Wey E, Githinji C, Rossor E, et al. The mental and physical health difficulties of children held within a British immigration detention center: A pilot study. Child Abuse & Neglect. 2009 Sep;33(9):573–85.
9. MacLean SA, Agyeman PO, Walther J, Singer EK, Baranowski KA, Katz CL. Mental health of children held at a United States immigration detention center. Social Science & Medicine. 2019 Jun 1;230:303-8.
10. Mares S. The Mental Health of Children and Parents Detained on Christmas Island: Secondary Analysis of an Australian Human Rights Commission Data Set. PubMed. 2016 Dec 1;18(2):219–32.
11. Mares S, Jureidini J. Psychiatric assessment of children and families in immigration detention - clinical, administrative and ethical issues. Australian and New Zealand Journal of Public Health. 2004 Dec;28(6):520–6.
12. Medecins Sans Frontieres. Indefinite Despair: The tragic mental health consequences of offshore processing on Nauru. [Internet]. 2018 [cited 2024 Jul 14]. Available from: https://msf.org.au/sites/default/files/attachments/indefinite_despair_4.pdf
13. Nielsen SS, Norredam M, Christiansen KL, Obel C, Hilden J, Krasnik A. Mental health among children seeking asylum in Denmark – the effect of length of stay and number of relocations: a cross-sectional study. BMC Public Health. 2008 Aug 19;8(1).
14. Reijneveld SA, de Boer JB, Bean T, Korfker DG. Unaccompanied Adolescents Seeking Asylum. The Journal of Nervous and Mental Disease. 2005 Nov;193(11):759–61.
15. Rothe EM, Castillo-Matos H, Busquets R. Posttraumatic stress symptoms in Cuban adolescent refugees during camp confinement. In Adolescent Psychiatry, V. 26 2013 Jun 17 (pp. 97-124). Routledge.
16. Rothe EM, Lewis J, Castillo-Matos H, Martinez O, Busquets R, Martinez I. Posttraumatic Stress Disorder Among Cuban Children and Adolescents After Release From a Refugee Camp. Psychiatric Services. 2002 Aug;53(8):970–6.
17. Sourander A. Behavior Problems and Traumatic Events of Unaccompanied Refugee Minors. Child Abuse & Neglect. 1998 Jul;22(7):719–27.
18. Sourander A. Refugee families during asylum seeking. Nordic Journal of Psychiatry. 2003 Jan;57(3):203–7.
19. Steel Z, Momartin S, Bateman C, Hafshejani A, Silove DM, Everson N, et al. Psychiatric status of asylum seeker families held for a protracted period in a remote detention centre in Australia. Australian and New Zealand Journal of Public Health. 2004 Dec;28(6):527–36.
20. Tosif S, Graham H, Kiang K, Laemmle-Ruff I, Heenan R, Smith A, Volkman T, Connell T, Paxton G. Health of children who experienced Australian immigration detention. Plos one. 2023 Mar 9;18(3):e0282798.
21. Young P, Gordon MS. Mental health screening in immigration detention: A fresh look at Australian government data. Australasian Psychiatry. 2016 Jan 11;24(1):19–22.
